# Counterparts

**Published:** 1887-06-18

**Authors:** 


### COUNTERPARTS.

By a Student of Eastern Philosophies.

(Continued from p. 183, vol. ii.)

### Chapter II.

When Edith found herself once more in a place of safety, she could not help feeling warmly towards her deliverer. She began to say some words of gratitude, when, looking down, her eyes fell on his feet, and she exclaimed in alarm, "Oh! what have you done to your foot? It is bleeding."

"I stepped on something sharp when I made that unlucky stumble," answered Paul. "Let it bleed! what does it matter?"

"It will matter if you get sand into it," remarked Edith, who privately thought he had not too much blood to spare; in which opinion she was wrong, for Paul had plenty of blood in his veins, though it did not show on the surface. "Come on to this rock and bathe your foot in the sea."

Paul hesitated a moment, then stepped on to the rock beside her.

"Give me your handkerchief," said Edith.

Paul obediently produced his handkerchief, but when she began to staunch the blood with it, he protested, and tried to take it from her. Edith, however, would not give it up, but after checking the bleeding, proceeded to fold her own handkerchief round his foot, in order, as she explained, to prevent the stocking from sticking to the wound; and so intent was she on her work, and so absorbed was her companion in watching her, that neither of them heard anybody approaching, till an angry and astonished exclamation of "Edith!" made them both start and look up.

Archie Bannerman, his handsome face flushed, his blue eyes flashing, and his soft, full lips trembling with jealous rage, stood before them.

"Why, Archie!" said Edith, in some trepidation, "I thought you were not coming till the seven o'clock train."

"So it appears!" replied Archie, looking, if possible, still more apoplectic than before.

Edith had regretted her unwise speech the moment it was uttered, but Archie always made her nervous when he took a jealous fit. He was certainly a formidable-looking object, as he stood there on the sands, panting like a bull of Bashan, his whole figure dilated with wrath and fury. There was no doubt about Archie's blood: it showed on every part of his surface which was not covered by hair or Scotch tweed.

"I mean," said Edith, trying to mollify him, "I mean, if I had known you were coming just now, I should have gone to the station to meet you."

But Archie refused to be mollified.

"What are you doing?" he demanded harshly.

"I am tying up this gentleman's foot, which he got hurt while saving me from being drowned—or at least drenched."

"How can you allow her to do such a thing for you?" asked Archie, turning fiercely to Paul.

"My permission was not asked," answered Paul, dryly.

"Nonsense, Archie!" exclaimed Edith, growing impatient. "To hear you, one would think that cut feet were infectious."

Archie growled.

"There, it is finished now," said Edith, rising and holding out her hand to Paul, which she would perhaps not have done had it not been for Archie's rudeness. "I hope it will not hurt you much when you walk. Good-bye—and thank you."

Paul bowed over her hand in silence, but as he looked earnestly into her face, it seemed to Edith as if there had passed from his eyes to her a something which vibrated like an electric shock through her whole system. Fortunately, Archie noticed nothing, though the parting glare which he gave his enemy as he raised his hat, could not, in any case, have been much more savage than it actually was.

In order to account for Archie's seemingly unreasonable ebullition of temper, it must be explained that Paul and he were not entire strangers to each other; they had met in London, and though scarcely more than three words of conversation had passed between them, so strong was the mutual antagonism of their natures, that each at the first glance had recognised in the other his bitterest foe. It was, indeed, hardly to be expected that two such diametrically opposed characters should have any sort of sympathy with one another; but why Paul's dislike to Archie, of whom he knew nothing, should be so largely mingled with contempt, and why Archie should feel tempted to break the sixth commandment every time his eye fell upon Paul, is as unaccountable on purely materialistic principles as it was decidedly unchristian. Possibly Archie's natural and unconscious insolence of demeanour, heightened as it would no doubt be by his contempt for any incomprehensible thing which could be called "quackery," made itself felt by Paul, in whom the graces of meekness and humility had not been highly developed, and whose natural resentment would so re-act upon Archie as to cause in the latter a deep regret that the days of duelling were over. Although both men had fallen in love, and that at first sight, with the same woman, their modes of treating this occurrence in their lives were characteristically different. Paul had surrendered himself without hesitation to the soul which he believed he saw looking out from the girl's dreamy, dark eyes. Archie, on the other hand, had struggled fiercely against the passion which mastered him. He knew it was foolish to grow fond of a girl who had neither the wealth nor the social rank for which he entertained so profound a respect; but though he acknowledged his infatuation, and his folly was ever before him, he was of too self-indulgent a nature to continue long in the attempt to keep out of her way. She amused him; her conversation was necessarily different from that of the other young ladies of his acquaintance, and he never felt bored in her company. Even before he engaged himself to her, he had a grand

King Cophetua-like feeling towards her, immensely pleasing to his vanity; but it is doubtful whether this would ever have declared itself definitely, had it not been helped by Archie's own passionately jealous temperament. Another man showed some attention to Edith: Archie flew into a rage; he vowed she should be his or nobody's, that he would shoot any man who came near her, and a great deal more to the same intent. The upshot was, that he presently found himself accepted by Edith, which rejoiced him exceedingly, though he lacked courage to tell his mother of his engagement. Lady Frances had her own suspicions, but she kept them to herself, like an experienced woman of the world, and quietly ignored the Charteris family—an easy thing to do, as they were very far removed from her "set."

Somewhat to Edith's surprise and disappointment, her lover had raised no objection to her leaving London. Archie was in fact rather relieved by her departure, as his position was beginning to grow a little embarrassing; but after the season had passed its height, and his time was no longer so fully occupied, he began to long for the society of his betrothed, and to find London very dull without her. He therefore determined to put off some unimportant engagements, and run down to Westborough for a day, just to make sure that his *fiancée* was not pining too much for him. It had never occurred to him that there were *men* at Westborough, or that any of them would dare to address Edith in his absence, and his chagrin was proportionately great when he found her apparently on such intimate terms with, of all men in the world, his enemy, Paul Travers.

Archie walked on for some time by Edith's side in a towering passion. He was really glad of a plausible excuse for his hatred of Paul, and very soon began to pour out his indignation upon him in words.

"What the dev—— I mean, what on earth has brought that quack down here?"

"What quack?" asked Edith.

"Paul Travers, of course; the man with whom you struck up such a sudden and violent friendship."

"Oh! is that his name?"

"Yes; unless you prefer the 'Wizard of the East,' or the 'Apostle Paul'; he gets various nicknames in town."

"But why do they call him the 'Apostle Paul'?"

"Because they say he pretends to cure people of their diseases by laying his hands upon them, and looking at them steadfastly. Do you mean to say you have never heard of him? Why, he was the rage in London just before you left."

"I do not know much about London 'rages,'" said Edith, apologetically; "we are not rich enough to go into society, you know."

"Ah! to be sure!" assented Archie, condescendingly; "but has he not been trying on any of his nonsense here?"

"I do not know; I never spoke to him until to-day."

"Oh!" said Archie, his brow beginning to clear; "but how came you to be speaking to him to-day? Who introduced him?"

"Nobody introduced him; I——"

"What! and you *spoke* to him?"

"Yes; very shocking, was it not? You see, I was surrounded by the tide, and he kindly helped me on to dry land; we could not do it all in utter silence, could we?"

Archie looked down at her dress.

"But you are not wet," he remarked. "How could you—you don't mean to say that he *carried* you?"

"Well, yes," answered Edith reluctantly; "there was not time to fetch a boat."

The vision of his betrothed in Paul Travers' arms was too much for Archie; he stopped in his walk and stamped on the ground.

"You must come back to London at once, Edith!" he exclaimed, furiously; "you are an abominable little flirt, and are not to be trusted alone."

"You know that is not true, Archie," remonstrated Edith; "you are most unreasonable. You don't suppose I liked to be carried, do you? Do stop scolding and let us be happy and comfortable during the short time you are here."

Archie stopped scolding, but relapsed into sulky silence, and refused to be happy or comfortable till after his ruffled temper had been soothed, and his inner man cheered by a hearty dinner. Then he regained his usual cheerful spirits, and the rest of the visit, which lasted till the following evening, when he took the train back to London, passed off peaceably enough; for Edith was particularly attentive and gracious to him, in order to make up for the annoyance she had inadvertently caused him.

Paul, in the meantime, having made the acquaintance of his "counterpart," in however unconventional a fashion, was determined not to let the grass grow under his feet. Accordingly, on Monday afternoon, with the handkerchief in his pocket as a pretext for calling, he presented himself at the door of Miss Charteris's house, and sought admission. Edith was out, but her aunt was at home, and Paul speedily proceeded to ingratiate himself with the old lady, which he did with so much success that she invited him to dinner, an invitation which he promptly accepted.

Edith looked grave when she came in and found how matters stood, but Paul's manner to herself was so perfectly unloverlike and matter-of-fact, that she was quite disarmed, and her mind set at rest. It was not till he had left that she remembered he had not returned the pocket-handkerchief to her. She concluded he had forgotten it.

After this Paul became a pretty constant visitor at Miss Charteris's house. The old lady had taken a great fancy to him, and frequently drew comparisons between him and Archie, seldom to the latter's advantage.

"I do not like young men who are not polite to old people," she would remark.

"I am sure, Auntie, Archie never was rude to you," Edith would reply.

"He never said anything actually rude to me; he merely ignored me entirely—in my own house too!" And Miss Charteris would blink through her spectacles and look very indignant.

Edith would then make a weak attempt to deny this assertion, but facts were against her, and she knew it.

"Edith," said Miss Charteris, suddenly, one day, "I

wish you were going to marry Mr. Travers, instead of that man."

Miss Charteris always spoke of Mr. Bannerman as "that man."

"My dear Aunt, he never asked me to marry him."

"No, of course not, because he knows you are engaged to that man; it is a pity you did not meet Mr. Travers first; I am sure he likes you."

"If I thought that, Aunt Mary, I should go back to London to-morrow, and never see him again. But it is quite a mistake; he never pays me the slightest attention beyond what any man would do." Edith honestly believed what she was saying, but she did not convince Miss Charteris, who merely remarked:

"I am no fool, Edith; I can see through my spectacles as well as most people, and allow me to tell you that you would be much happier as Paul Travers' wife than as Archibald Bannerman's."

"All right, Auntie; shall I break off my engagement with Archie and propose to Mr. Travers the next time he calls? It might be awkward, however, if he refused me after all."

Here Edith observed Mr. Travers approaching the house, and in a sudden fit of shyness she flew to her room, and locked the door.

After such a conversation it was impossible for Edith to be so much at her ease with Paul as before. She began to observe him more carefully than she had hitherto done, and noticed that although he did not speak much to her, he looked at her a good deal, and that if she went to the piano to sing or play, he always chose a seat whence he could watch her face. Those sombre eyes, with their hidden yellow glow, began to have a strange fascination for her. If she met their gaze it was with difficulty that she could withdraw her own, and she always knew, without looking, when they were fixed upon her. Edith began to be alarmed. Was he trying to mesmerise her? was there any truth in mesmerism after all? Archie said "No," yet——

Edith determined to sound Mr. Travers on the subject the very first opportunity that occurred.

*(To be continued.)*

